# Women’s experiences with the healthcare system during and after a late miscarriage: a qualitative meta-synthesis

**DOI:** 10.3389/fpsyt.2025.1673215

**Published:** 2026-01-05

**Authors:** Wictoria Kristiansen, Birgitte Mæhlum, Jens C. Thimm

**Affiliations:** Department of Psychology, UiT The Arctic University of Norway, Tromsø, Norway

**Keywords:** late miscarriage, healthcare, women’s experiences, meta-synthesis, meta-ethnography

## Abstract

**Introduction:**

Miscarriage is a relatively common pregnancy outcome and often imposes a significant psychological burden on those affected. Previous research suggests that the role of the healthcare system can greatly influence the psychological consequences of a miscarriage. Several literature reviews have addressed women’s experiences with the healthcare system in relation to early miscarriage. Late miscarriage, however, has received less attention in the research literature. The aim of this study was to synthesize qualitative literature on women’s experiences with healthcare in relation to late miscarriage.

**Methods:**

The study is a qualitative meta-synthesis based on a systematic literature review conducted across four databases (CINAHL, Embase, Medline, and PsycInfo). Eight qualitative studies, comprising a total of 95 female participants, met the inclusion criteria and were included in the meta-synthesis. The data were analyzed using a meta-ethnographic approach.

**Results:**

The analysis identified three interconnected main themes that center on validating women’s emotional and practical needs of the women: (1) Acknowledgment of the loss, (2) The power of language, and (3) Institutional factors. The women emphasized that healthcare professionals should recognize and validate their loss, demonstrate empathy and compassion and provide clear, comprehensible information delivered sensitively. Hospital facilities and other systemic factors also had a significant impact on how the women felt cared for and supported.

**Discussion:**

The study findings suggest areas for improving care and follow-up measures for those who experience late miscarriage. Further research on the topic could enhance understanding and contribute to the development of improved healthcare interventions for those affected.

**Systematic Review Registration:**

https://www.crd.york.ac.uk/prospero/, identifier#CRD42024584350.

## 1 Introduction

Miscarriage is a relatively common pregnancy outcome and can be a painful experience for those affected (1). The consequences of a miscarriage often include physical ailments but also entail a psychological burden, both in the short and long term (2, 3). The healthcare provided to women in connection with miscarriage is considered central to how they cope with and process the loss of a child (4, 5). Several qualitative literature reviews have been conducted on women’s experiences with the healthcare system related to involuntary termination of pregnancy occurring before week 12 [e.g., (4, 6)]. However, women’s experiences of the healthcare system in connection with late miscarriage are less researched. Thus, we aimed to summarize and synthesize existing qualitative research about women’s encounters with the healthcare system in connection with late miscarriage.

Miscarriage is broadly defined as the termination of pregnancy before viability (3). While there is consensus to label pregnancy loss before gestational week 12 as early miscarriage [e.g., (7)], the distinction between late miscarriage and stillbirth varies between countries. For example, in the US, pregnancy loss before the 20^th^ week is considered late miscarriage (8). In Scotland, on the other hand, late miscarriage is defined as a loss of pregnancy before week 24 (7). Miscarriages before week 20 of pregnancy are relatively frequent, with a prevalence of approximately 10-20% (9, 10), and the vast majority (80%) occurring during the first 12 weeks (11).

The cause of miscarriage is often unknown but may be associated with problems related to the fetus, uterus/cervix, hormone production, chromosomal defects, or infection (10). Advanced maternal age is a key risk factor for miscarriage, which is more common in women over the age of 30 and with the highest prevalence in women over the age of 45 (75%; 12). Furthermore, an unhealthy lifestyle, obesity, and previous stillbirth or miscarriage can increase the risk of involuntary termination of pregnancy (3).

A miscarriage is associated with adverse health outcomes, including an increased risk of both perinatal death and preterm birth in later pregnancies, especially after a late miscarriage and multiple miscarriages (2, 3). Furthermore, an increased risk of fetal growth restriction, premature placental abruption, and stillbirth has been found in later pregnancies (3). Women who have undergone one or more miscarriages also have an increased long-term risk of cardiovascular disease and blood clots (3).

Grief is a common reaction after a miscarriage (13, 14). Research findings suggest that the intensity of parental grief increases with the age of the fetus (15). This is probably in part due to the fact that the mother’s attachment to the fetus strengthens with the length of the pregnancy (16, 17). Many women experience that their grief in connection with the involuntary loss of pregnancy is not recognized and validated by others (18, 19), which can add to the psychological burden.

Beyond grief, miscarriage can be associated with a number of other negative psychological outcomes for both the women and their partners. Studies have shown that women who have had a miscarriage are more likely to report symptoms related to mental health problems, such as anxiety, depression, and post-traumatic stress disorder (PTSD; 20–23). Although there is generally a decline over time, persistent symptoms of mental health problems have been found three or more years after the loss (24). Women who underwent a miscarriage are also at increased risk of self-harm and suicide (22).

In addition to the physical and mental health outcomes, miscarriage and the loss of a child can have an impact on women’s interpersonal and sexual relationships, as well as their identity (25). The experience of social isolation after a miscarriage has been shown to be positively correlated with symptoms of PTSD (26). Following the loss of a child, parents also report changes in their relationship, such as a decline in sexual intimacy, a decrease in the sense of community, and difficulties with both everyday and emotionally charged conversations (27). Similarly, Tian and Solomon (28) found that women’s negative perceptions of their miscarriage were positively correlated with relational insecurity. Moreover, identity can also be affected by miscarriage. For example, some women experience a change in identity from “woman” to “mother” when they become pregnant, and for these women, undergoing a miscarriage can be experienced as a major identity challenge (29). Women who have undergone a miscarriage also report that the feeling of motherhood has become more important to them (30).

Satisfaction with the healthcare received in connection with a miscarriage can have an impact on the psychological distress experienced afterwards (31). Satisfaction with healthcare provision and information about physical changes after a miscarriage has been shown to be associated with less perinatal grief and depression (5, 32).

Qualitative studies have highlighted that women who experience miscarriage often report inadequate healthcare related specifically to the hospital environment and medical management, both during and after the event (31). In contrast, after a stillbirth, parents often experience adequate care and support during the hospital stay but insufficient aftercare after discharge (33). Furthermore, men’s and women’s emotional well-being after a miscarriage is linked to interactions with healthcare professionals, information provision, and hospital environment, specifically concerning the time of diagnosis and treatment, lack of privacy in the hospital setting, recognition of the loss, and the use of medical terminology (6). These findings coincide with a recent review (4) that found that women who have had a miscarriage highlighted three main aspects: a preference for patient-centered care in the form of clear communication and empathy, the experience of an over-medicalized approach, which in turn leads to poor communication, and improvement of the patient experience by informed decision-making.

Taken together, existing research studies suggest that miscarriage is associated with both physical and psychological challenges and that healthcare professionals’ approaches and systemic factors can have a major impact on how those affected feel met and cared for. Limitations of previous reviews of the research literature include not distinguishing between early and late miscarriage (6), focusing exclusively on the U.S. population (4), or to a lesser extent, giving limited attention to the role of the healthcare system (34). However, research suggests that the bonding with the fetus is stronger later in pregnancy, and that the intensity of grief also increases in connection with miscarriage late in pregnancy (15). This may suggest that the healthcare needs of women who undergo late miscarriage and their experiences of their encounters with the healthcare system may be different from those who experience early miscarriage. Therefore, the purpose of this study was to review and synthesize the qualitative literature about women’s experiences with healthcare in connection with late miscarriage, and the research question was: How do women experience their encounter with the healthcare system during and after a late miscarriage?

## 2 Methods

### 2.1 Choice of methodological approach

In this study, we chose to use qualitative meta-synthesis with a meta-ethnographic approach, as described by Noblit and Hare (35). The aim of a meta-ethnography is to synthesize findings from previous qualitative studies to create a new understanding of a topic or phenomenon (36). We chose a meta-ethnographic approach because of its unique way of interpreting human phenomena and experiences, and the possibility of drawing lines between different studies (35). Furthermore, this method is particularly well-suited for developing concepts and models from its analytical approach, where it differs from other approaches of a more descriptive nature. The aim of meta-ethnography is to interpret the primary authors’ original interpretations while using the participants’ own statements to support these interpretations. Thus, it builds on the original findings of the individual studies to provide a higher-order interpretation of the results (37). This distinguishes meta-ethnography from other qualitative synthesis approaches, such as thematic analysis, which does not generate an interpretive interpretation, but rather creates a more descriptive interpretation of the data material (38).

Noblit and Hare’s (35) method consists of seven phases: 1) getting started; 2) deciding what is relevant considering the problem; 3) reading the studies; 4) finding connections between the studies; 5) translating the studies into each other; 6) synthesizing the translations; and 7) presenting the synthesis. These phases are worked on in an iterative process, i.e., they overlap, and one moves between the phases (39). The work on the first six phases is presented in the following. The seventh and final phase is presented in the results section.

We acknowledge that our perspectives may influence the selection and interpretation of literature. The study is based on the two first authors master’s thesis in psychology, which was supervised by the third author. We recognize that our training as psychologists and Western cultural context can shape how we interpret women’s narratives. To address this, the selection of the studies was conducted independently by WK and BM, and consensus meetings were held to resolve disagreements. The coding and the synthesis of have been done jointly to minimize bias.

### 2.2 First phase: getting started

This phase concerns finding a field of interest and a knowledge gap in the literature (35). The aim of the current study and the rationale for using meta-ethnography are stated above. A protocol for pre-registration in the International Prospective Register of Systematic Reviews (PROSPERO, ID: CRD42024584350) was submitted on August 30, 2024. Deviations from the pre-registered protocol are described below.

Together with a university librarian, we decided on the selection of search terms and search engines. We used CINAHL, Embase, Medline, and PsycInfo and different filters to search for qualitative studies in these databases, based on the recommendations by Rogers et al. (40). This was to achieve the best balance between specificity and sensitivity in each database. We applied the filter from the University of Texas for Medline, Roger et al.’s (40) filter for PsycInfo, the Wilczynski(f) (41) filter for CINAHL, and the CHLA filter for Embase. We then combined the results from the search for miscarriage and the results from the search for qualitative studies with “AND” (see **Supplementary Table S1** in the online **Supplementary Material** for an example of a search string in PsycInfo). Initially, we wanted to include “women” in the search string in order to exclusively include women’s perspectives. After conferring with the university librarian and conducting a test search, we found that this resulted in too few search results. Therefore, we chose to exclude “women” from the search string to ensure that the search was broad enough to include all relevant articles.

**Figure 1:**
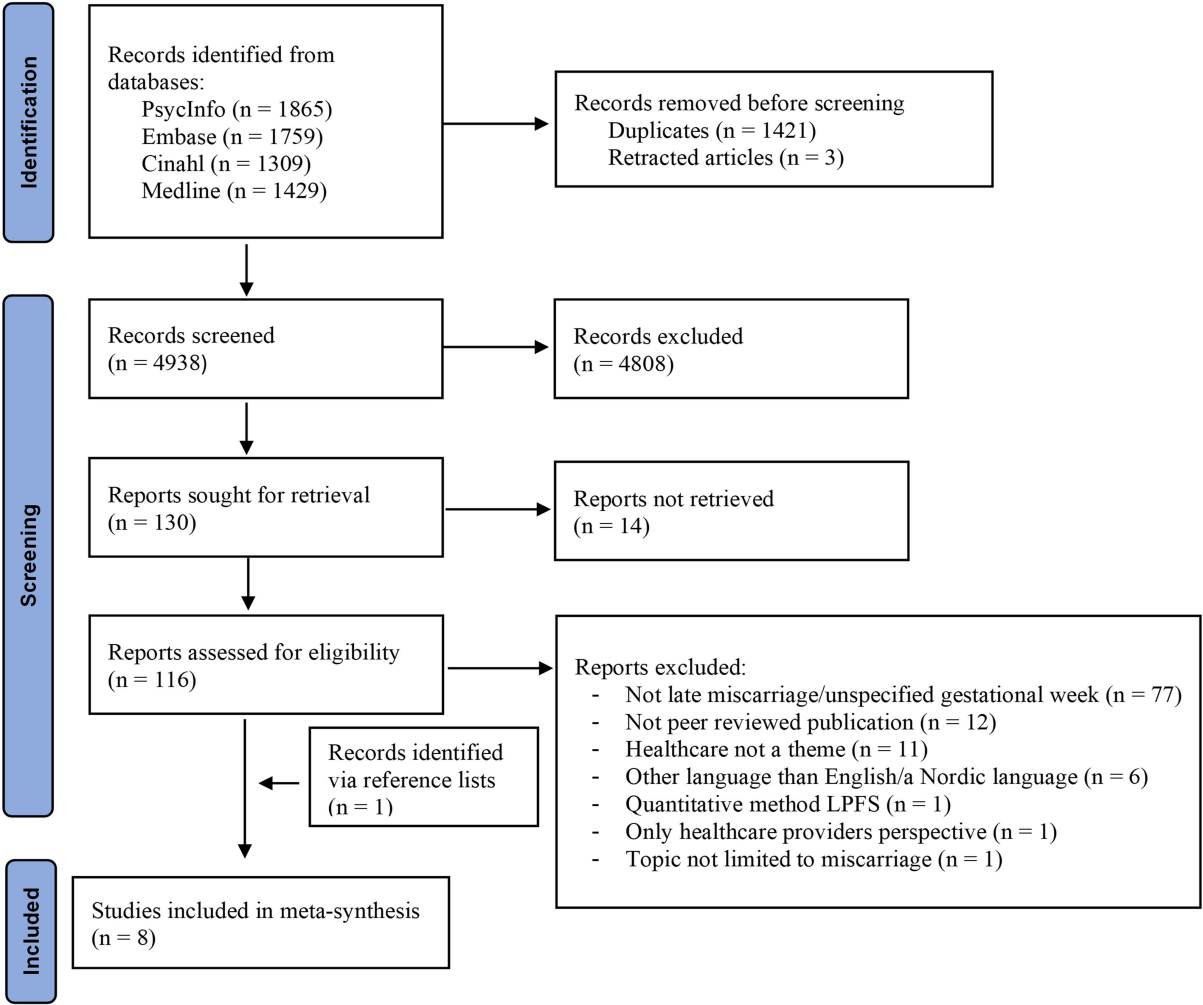
PRISMA flowchart illustrating the study selection process. Note: Flow chart template retrieved from Page et al. (42).

We conducted the search on September 3, 2024, yielding 6362 publications. The search results were downloaded to EndNote 20. After removing 1421 duplicates, we were left with 4941 studies. Three of these were registered as retractions, which left us with 4,938 studies to review in title and abstract (see **Figure 1** for an overview of the entire selection process).

### 2.3 Second phase: deciding what is relevant considering the problem

#### 2.3.1 Definition of late miscarriage

Due to the many different definitions of late miscarriage, we decided to apply a broad definition: miscarriage between 12 and 24 weeks of pregnancy (7). There are fewer studies on this phenomenon than on early miscarriage, probably because late miscarriage has a significantly lower prevalence. Therefore, a broad definition of late miscarriage was used to include a larger number of studies, strengthening the empirical basis of the synthesis.

#### 2.3.2 Selection based on broad criteria

We initially defined some broad inclusion criteria. These criteria were that the articles should be qualitative, focus on late miscarriage, and include women’s perspectives. Studies that used mixed methods, were restricted to early miscarriage or stillbirth, or contained mainly healthcare professionals’ or only men’s perspectives were excluded. This screening was conducted independently by WK and BM. We used the tool Rayyan (https://www.rayyan.ai/) to screen the search results. After resolving conflicting assessments, we identified 130 articles to be reviewed in full text.

#### 2.3.3 Selection based on narrow criteria

The process of reading the articles in full text was also done separately by WK and BM. The narrow criteria for inclusion were as follows: more than 50% of the participants in the study had a late miscarriage (between 12 and 24 weeks), more than 50% of the participants were women, the experiences with the healthcare system related to the miscarriage were mentioned, and publication in a peer-reviewed journal. A quality assessment of the studies based on the criteria in the Critical Appraisal Skills Program (43) was conducted.

A total of eight articles were included in this meta-synthesis. We initially included seven articles after reading the full text and found one more article after reviewing the reference lists of a similar study (34). The characteristics of these studies are presented in **Table 1**. The quality assessment using CASP suggested that all eight studies had sufficient quality to be included in the synthesis (see **Supplementary Table S2** in the online **Supplementary Material**).

**Table 1:** Overview of included studies.

Study	Country	Aims	Participants	Data collection	Analysis
Corbet-Owen & Kruger (44)	South Africa	To determine the impact of pregnancy loss on women, to explore their emotional needs in the aftermath of the loss, and to assess whether and to what extent healthcare professionals recognize and address these losses	Eight heterosexual women aged 18–33 years at the time of the loss(es) who had experienced 1–4 losses	Open-ended interviews with specific additional questions about interactions with healthcare professionals	Constructionist grounded theory techniques
Cullen et al. (45)	Ireland	To explore parents’ experiences of clinical care after second trimester pregnancy loss, from diagnosis to follow-up	Nine mothers (age 30–42 years) and five fathers contacted 6–24 months after a second trimester miscarriage. Five mothers had previous miscarriages.	Semi-structured interviews, conducted separately with mothers and fathers, except for one couple	Thematic network analysis
Ekelin et al. (46)	Sweden	To conceptualize women’s and partners’ experiences and coping strategies before, during, and after a second trimester ultrasound with nonviable fetus as diagnostic outcome	Nine women aged 28 to 40 years and six partners. One couple had a previous miscarriage. Participants were recruited from a hospital’s ultrasound department. Interviews were conducted 1.5 to 9 months after the examination.	Open-ended interviews	Grounded theory
Kukulskiené & Žemaitiené (47)	Lithuania	To investigate experiences of late miscarriages and describe practical implications for postnatal healthcare	Seven women aged 29 to 52 years recruited via “snowball” sampling. Participants had experienced 1–3 miscarriages.	In-depth interviews	Phenomenological thematic analysis
Lee (48)	Australia	To describe Australian women’s experience of late pregnancy loss, with a focus on their experiences in healthcare	14 women aged 23 to 39 years contacted 3–4 months after the loss. One participant had a previous miscarriage.	Open-ended, digital survey	Phenomenological thematic analysis
Mulvihill & Walsh (49)	Ireland	To understand pregnancy loss through a constructivist epistemological approach	Eight women aged 30 to 42 years with 1–5 pregnancy losses.	Interviews	Interpretive phenomenological analysis
Sanchez (50)	USA	To investigate mothers’ perceptions of support from hospital-based health professionals following a perinatal loss	12 women aged 30 to 44 years recruited from a university hospital.	Semi-structured interviews	Unspecified
Smith et al. (51)	United Kingdom	To explore parents’ experiences of healthcare after a pregnancy loss between week 20 and week 24 of pregnancy	38 parents (10 parent pairs and 18 mothers) recruited through parent support organizations and clinicians involved in the study.	Semi-structured narrative interviews	Modified grounded theory

#### 2.3.4 Decisions in the selection process

At the start of the selection process, we initially intended to include only recent studies from 2018 onward, but we quickly realized that this was not sufficient if we wanted a comprehensive data material. We did not exclude studies based on the healthcare context in which the study was conducted (hospital, ultrasound examination, unspecified) or the qualitative method used (interview, open-ended survey) as long as the article itself was of adequate quality. We only included articles that contained women’s perspectives, thus excluding the perspective of healthcare professionals, as our aim was to highlight the voices of the women who had undergone the miscarriage. To obtain a richer data set, we also included studies that included both the women’s and their partners’ perspectives and omitted the quotes from the partners during the coding and synthesizing process. We also decided to exclude doctoral theses, case studies, and other grey literature. This is because such literature does not necessarily have the same formal requirements as peer-reviewed articles, which in turn can affect both scientific and quality standards.

### 2.4 Third phase: reading the studies

In this phase, WK and BM read the studies together and divided them into meaningful units. These meaningful units consisted of quotes (first-order constructs) and descriptions/metaphors from the authors (second-order constructs), which were interpreted by us and given appropriate analytical codes. An example of how the units were divided is shown in **Table 2**. Disagreements related to the formulation of codes were discussed until agreement was reached. In some cases, there were only quotes from fathers in the study, which were not analyzed. However, we used the authors’ interpretation if it was indicated that it applied to both men and women. Interpretations or quotes that did not refer to experiences with the healthcare system or expressions of needs or changes from healthcare professionals or the healthcare system were also coded to preserve the context.

**Table 2:** Example of how the units were divided and analyzed.

Primary authors’ interpretation	Quote	Analytical codes
Facilities and privacy: A negative experience of being cared for in a multibedded public ward was also described. Participants stressed the importance of respecting privacy at such an emotionally difficult time.	*“They wanted me to go into a commode with a ward full of five other people with visitors … I thought there was no humanity to it all … very undignified … a horrible experience” (Sarah, 12/40)*	Facilities, organization, privacy

### 2.5 Fourth phase: finding connections between studies

The meaningful units were uploaded into the NVivo 15 software, where the analytical codes were entered. After completing the third phase, we already had an idea of which themes were important and which themes were recurring across the various studies. In the fourth phase, we therefore worked further on this categorization by comparing the themes in light of our own codes and looked for similarities and differences in the data material (cf. 35). In our data material, we mainly found similarities.

### 2.6 Fifth phase: translating the studies to each other

This phase involves systematically comparing the similarities identified in phase four using reciprocal translation synthesis. This involves understanding the studies in light of each other through their metaphors and concepts and finding themes that explain these across multiple studies (35). To identify these associated overarching themes, we discussed concepts that fit the quotes and the primary authors’ and our own interpretations (cf. 39). If a code could be interpreted in more than one way, the original article was retrieved to discuss which interpretation was most descriptive of the data. We started with 236 unique codes, which were sorted into main categories through discussion. In the end, we concluded with three overarching codes that covered the data material in the synthesis. These three overarching codes also had several sub-codes that formed the basis for the sub-themes of each main theme.

### 2.7 Sixth phase: synthesizing the translations

To interpret the three main codes, we analyzed the sub-codes and their quotes, identifying theme labels to define the overall phenomenon. We emphasized the most comprehensive sub-codes and how these could cover the essence of the main code. This step is considered the core component of Noblit and Hare’s (35) method, where the synthesis should go beyond the sum of its parts and contribute something more than what the parts alone can contribute. This resulted in our third-order constructs, which are presented in the results section.

## 3 Results

**Figure 2:**
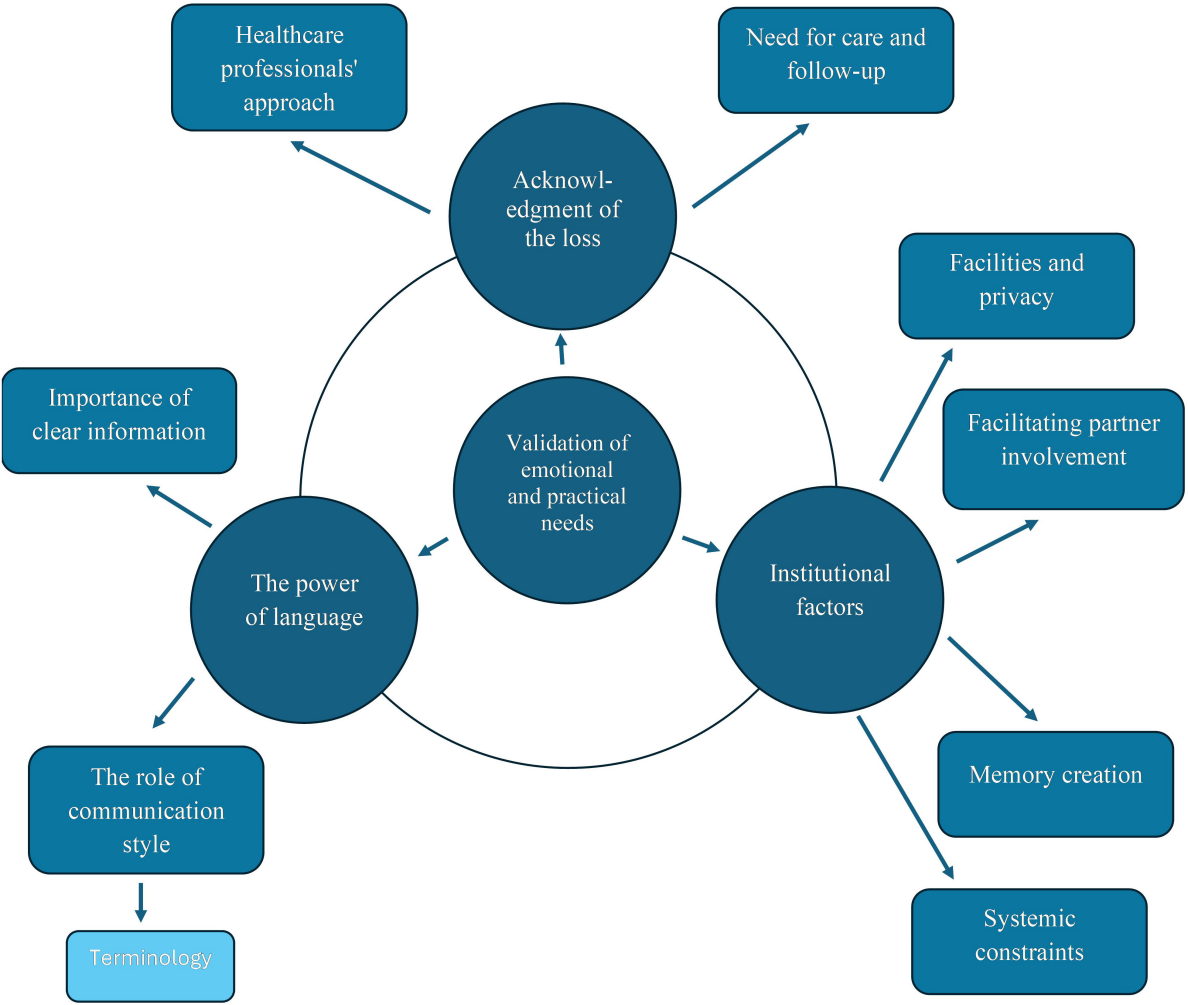
Conceptual model illustrating the connection between the themes in the analysis.

The analysis identified three key themes: *1. Acknowledgment of the loss, 2. The power of language*, and *3. Institutional factors.* Under the first main theme, we found the sub-themes *1.1 Healthcare professionals’ approach* and *1.2 Need for care and follow-up*. Under the second main theme we found the subthemes *2.1 The importance of clear and complete information* and *2.2 The importance of the role of communication style*. We also found a subtheme *2.2.1 Terminology*. Under the last main theme, we found the sub-themes *3.1 Facilities and privacy*, *3.2 Facilitating partner involvement*, *3.3 Memory creation*, and *3.4 Systemic constraints*. These themes overlap and mutually influence each other but are nevertheless understood as qualitatively different. A common core across these themes is validation - i.e., the empathic understanding and legitimization of women’s emotional and practical needs following a late miscarriage. Validation is therefore at the center of the conceptual model of the study findings and proposed to underlie the three main themes and their subthemes (see **Figure 2**). The themes are presented below with our interpretation of the findings, illustrated by direct quotes reproduced from the individual studies.

### 3.1 Acknowledgment of the loss

One of the first themes that became clear during the analysis was the need for acknowledgment of the loss. This theme manifested in different ways for the women. It was primarily about the need for care and follow-up in the grieving process, which many women felt was lacking: “I asked about that help. I asked where I could get professional help. Well, you had just given birth to your dead, ummm, child. I don’t know. So, they said there was none” (“Ko”, 47, p. 12).

#### 3.1.1 Healthcare professionals’ approach

Many of the women had experiences around the miscarriage where the emotional impact was significantly influenced by the healthcare professionals’ approach. The emotional impact of the miscarriage seemed to be largely shaped by healthcare professionals’ handling of the situation and their ability to demonstrate care and empathy. An important aspect highlighted by several participants that could hinder the experience of validation and empathy was that they sometimes felt that healthcare professionals had a different perception of the procedure than they did:

(…) felt “patientized,” if I could say so. I was just an ordinary case. You know, someone was going to be operated on appendicitis, I was supposed to push the child out, yet somebody else had to have their burnt leg bandaged. Oh, of course, the sterility differed, but in essence, everything was the same. (…) your leg will heal, you haven’t lost anything, (…) but this is still a part of your body, not some other person in you. (“Ko”, 47, p. 7).

This quote describes how undergoing a miscarriage was compared to other types of “ordinary” interventions, which several participants perceived as inappropriate or invalidating. This could give the women a sense that their grief and loss were not given the recognition they needed.

It was also painful for participants when they felt that healthcare professionals did not treat them with kindness and compassion. “Jackie” highlighted, among other things, how a caring approach from healthcare professionals could have improved her experience: “I can’t really put any blame - other than the insensitivity from that nurse and maybe the doctor … he was so offhand … it would have helped to have a caring doctor … Nurses need to be VERY sensitive” (44, p. 421). At the same time, several participants described that being met with recognition had a significant impact. For example, “Belinda” described the experience as positive after being shown respect and treated with dignity, even though it was a stressful experience initially:

They were really wonderful. I think everyone was being extra nice to us due to our circumstances but we actually came away feeling like we had a somewhat positive experience - after all it was the birth of our first children and they made it feel just like that. (…) We didn’t know what to expect having been rushed into the experience unexpectedly, but it felt as though the birth of our babies was treated with the respect & dignity of any normal birth - maybe even more so. (“Belinda”, 48, p. 69).

The importance of sensitive and empathetic care was consistent across several of the studies, and many of the women also expressed how a supportive approach from healthcare professionals could have an impact on their experience:

I had met the 2 midwives previously. The midwife who discovered our terrible news with me was there through the entire birth and was so kind and supportive. The other was there at the start of the labor induction and was back in the morning and collected all the little bits and pieces for our little memory boxes. Everyone was extremely kind and supportive (“Belinda”, 48, p. 68).

The quote further illustrates the value of continuity of healthcare professionals, which was pointed out by several participants. This was linked to the strong emotional experience that led to a need for security in the form of familiar staff.

At the same time as sensitive and empathetic care was central to those who experienced a profound loss, it was also important for those who did not experience the miscarriage as great loss or had a need for closure to be met with understanding. “Lisette” expressed “I did not want to see the baby or to touch her so I felt better not to see her” (48, p. 68). This illustrates how the experience is individual, and that it should not be taken for granted that there is a desire for personification or memory creation, a topic we elaborate on below.

“Sandy” explained that she only wanted to forget the event: “I didn’t want to speak about it … I just wanted to take a rubber and erase it out of my life” (44, p. 422) and went on to say “he thought I was having a great loss … he didn’t know my true feelings … he didn’t know that it was a relief in my life…” (p. 419) about the doctor who tried to comfort her. This shows that the loss does not have to be equally stressful for everyone, and that not all participants necessarily received the same validating effect from the approach taken by healthcare professionals. One of the women in Sanchez’s (50) study said about the pictures taken of the child that ‘‘those photos were too clinical’’ (p. 28). These quotes illustrate how the feelings that arise after a late miscarriage can be different, but that the need for validation and understanding remains.

#### 3.1.2 Experiences and needs around follow-up

Several participants in the studies expressed a desire for follow-up after the miscarriage, and although the focus of this follow-up varied, the need for such an offer applied to several. One of the women in the study by Ekelin et al. (46) said “But we kind of needed something to finish it off” (p. 451) in connection with the fact that they were not offered a follow-up conversation or contact with a counselor by the hospital. Another participant suggested that there should be more focus on the emotional follow-up to have a kind of safety net in the grieving process:

Maybe after some six weeks, when you have to go to the doctor. Then, one way or another, maybe by asking how we were feeling, they would see whether there was some depression or that perhaps something was coming; that you just couldn’t go through this, that you were still very distressed, very upset. Then maybe they would direct you to someone else. (“J”, 47, p. 12).

Many women wanted to be followed up by the hospital. It didn’t necessarily have to be continuous, but several women felt that they needed to talk to someone about the feelings they were experiencing and that the follow-up itself also had a validating effect on the loss and grief: “Shows that you’re not just a number, you’re a person and you’re going to be looked after” (“Monica”, 49, p. 2298).

Furthermore, the lack of follow-up during or after the procedure was described as a painful experience: “I was alone [sniffles], there was no one I could, I mean, nobody supported me [with a breaking voice, cries out loud, a long pause, sniffles, a long pause]. I was sitting in the corridor [in hospital] crying” (“M”, 47, p. 7). One of the participants also pointed out the need to not have to ask for this follow-up, but that it should be offered in the first place: “It’s hard to ask for support isn’t it … I think it’s easier to accept the offer than to go look for it yourself” (“Bernie”, 49, p. 2298).

### 3.2 The power of language

The importance of information was significant for many of the participants, both during and after the miscarriage. This meant that information needed to be clear, timely, and provide the mothers with an explanation of what had happened. The way healthcare professionals communicated was also crucial to how the women felt cared for.

#### 3.2.1 The importance of clear information

Open, concrete, and direct information could help the participants feel a sense of control and security. Detailed explanations during the process were viewed positively by several mothers, e.g., “Libby”: “He totally, he analyzed the whole physical thing … what had gone wrong and you know why it had happened and all the questions that we asked, he explained everything…” (44, p. 420). “Kate” also explained that, although it was difficult at this time, it helped her in retrospect that the doctor had been clear during the actual hospitalization: “We were told at 12 weeks. He was very clear about … He was very black and white about things, which I found hard at the time, but I much appreciated later on” (45, p. 313). Other participants, however, expressed that a lack of information could lead to the feeling of being left with questions about whether the miscarriage could have been self-inflicted: “There is no explanation … all of those questions inside of me … where have I failed?” (“Nicky”, 44, p. 421).

“Nicky’s” quote illustrates another important aspect of information that was prominent in several of the studies, namely giving the mothers an explanation of why the miscarriage had happened to help avoid feelings of guilt and shame. For example, “Fiona” expressed that it would probably have been easier to deal with the event if she had been given clearer information about the reason for the miscarriage: “The inability of doctors in some cases to offer a clear explanation for the loss was particularly hard: Still I think if I knew there was a reason, I think it would be easier to live with it” (49, p. 2296).

A third aspect that several participants considered as crucial was the timing of the information provided. Several women mentioned the importance of receiving information in advance of procedures in order to be able to plan and prepare better for what they were about to undergo, which this participant expressed frustration about:

I wasn’t given an appointment and told “There’s no hurry” and “It will probably come out by itself.” I felt very, very disappointed that I couldn’t be given some kind of info and not even be given an appointment. If I had only known that it’s on Friday, even if that was a week away, I would have known that it would be then. That would have made me feel better. I went to the emergency department anyway. I think it was on Monday evening. I just felt I somehow had to force my way in. (46, p. 450).

Another recurring theme in several of the studies was information that the participants considered unnecessary and inappropriate at a time when they were in a crisis characterized by grief and despair. This included information about prognoses for a possible next pregnancy: “[The doctor] says, ‘Recommendations regarding conception- next time.’ And I’m just thinking to myself that right now I’m not interested in any conception at all” (“J”, 47, p. 10).

Other women felt that healthcare professionals were more concerned with providing information about financial rights, when the women really needed care and support in connection with the loss:

… maybe an hour after we had had our scan … saw a social worker who was very rude I felt. She had no emotion and basically said this happens all the time and that we can get the baby bonus [a family assistance payment]. Who wants to hear that when they have just been told that they won’t have a baby to take home!!! Very insensitive (“Anna”, 48, p. 66).

#### 3.2.2 The importance of communication style

Another important aspect of communication was the way information was conveyed: “Just the way they spoke, their approach, I suppose it wasn’t what they said, but how they said it” (“Bernie”, 49, p. 2295). Most expressed a need for a careful approach, and it often emerged that an empathetic communication style was preferable as they were already in a very vulnerable situation:

… The one incredible strength about the gynecologist is that he never downplays the emotional side … he kept saying to me: ‘Just deal with your emotions. Don’t let anyone tell you that this is the right way or this is the wrong. Your emotional health is as important as your physical health’” (“Libby”, 44, p. 418).

At the same time, not all communication was characterized by care and empathy. “Libby” goes on to explain how she was also met with a lack of respect and understanding from other healthcare professionals at the start of her hospitalization:

My initial experience … was hideous … TOTALLY unsympathetic … start(ed) screaming and shouting at me to calm down and control myself otherwise I would CAUSE the miscarriage … Nightmare. (44, p. 421)

Another recurring theme in the studies was comments from healthcare professionals that could indicate a lack of understanding of what the women were experiencing. The use of humor and irony was also not appreciated by several women and was seen as insensitive and a lack of empathy:

He said, “Oh, it’s like winning a million in a lottery.” Yeah, lucky, but unlucky. And I just thought to myself, “How can you possibly compare this to some lottery” [in a raised voice, laughs], (…) So, I said that maybe it was high time to start looking at what could be done specifically, that maybe we should do tests instead of playing a lottery … I remember I didn’t like it at all and I never went to him again. (“J”, 47, p. 10)

Other women were met with seemingly well-meaning comments that were experienced as inappropriate given the context and timing:

A doctor came and talked with me while I waited for my family to arrive and while I think she was trying to be sympathetic, she was telling me about how she had had several miscarriages before having her own children, so always hope etc. - and this was not the time to be talking about this with me. (“Galia”, 48, p. 65)

##### 3.2.2.1 Terminology

Several of the participants across the studies expressed frustration with the language used by healthcare professionals. Many felt that they didn’t understand what was being talked about, which could lead to more confusion and anxiety in an already stressful and vulnerable situation:

… we had no idea what the doctor was talking about as we had never heard of it [anencephaly]. All I remember the doctor saying to us was NOT COMPATIBLE WITH LIFE (…) It wasn’t a very nice time and we were so upset. (“Nadine”, 48, p. 65)

However, another participant explained that using few and understandable words, combined with a careful approach, created a sense of being understood and cared for:

Simply, in simple words, directly admitting that those were the most painful situations at work for her as well; and she did show interest in me asking about my previous experiences, well, simply caring about me. I do remember such episodes from our conversations. Very simple language, really, but … I felt cared for. (“S”, 47, p. 11)

Furthermore, the use of different terms and unclear definitions around pregnancy loss had an impact on several participants. Some women felt little recognition of the loss when healthcare professionals used words such as “miscarriage” rather than, for example, “losing a child”. “Camille” underwent pregnancy loss in week 21 and felt using the word “miscarriage” did not acknowledge the loss or adequately prepare her for what she was about to go through: “Being told ‘you’re having a miscarriage’… it doesn’t prepare you for it” (51, p. 870). In addition, several women noted that healthcare professionals referred to the baby as a “product” or a “thing”, which was considered impersonal and demeaning: “And you know the terms they use ‘the products of conception’, that’s very insensitive for parents to hear that” (“Sarah”, 49, p. 2297).

For several women, a more appropriate use of terminology was helpful in dealing with the loss afterward, as expressed by “Sarah” here:

I knew a lot of other people who’d had babies similar time as us, similar week, but had been treated - you know - not badly, but they felt that they had been treated as if they’d had a miscarriage’ and this made a ‘massive difference to how we dealt with it afterwards. (51, p. 871)

### 3.3 Institutional factors

A final prominent theme was the institutional factors. This theme related to how the hospital environment and practical arrangements had an important impact on the experience the women were left with and whether they felt their loss was met with dignity and taken seriously.

#### 3.3.1 Facilities and privacy

This theme focused on how the women experienced hospital facilities, and whether they felt they had privacy. “Michelle” described having to leave the room to go to the toilet as very difficult: “I would have had to go to the toilet just across the way … it was really really difficult … I suppose there isn’t much of a choice but there should be” (45, p. 313). The intense emotional pain they went through was also difficult to reconcile with a double room and could be embarrassing for both the women themselves and other patients they shared a room with: “I was brought back to the ward and I couldn’t sleep, I was just sobbing. And there was this other woman, and I was worried that I might wake her up because the pain and grief were profound” (“K”, 47, p. 13).

Many of the women also pointed out a need to be separated from other pregnant women and newborn babies, as this became a painful reminder of the loss: “I know there really isn’t anywhere else for Mums who have lost or are losing their babies but it really is awful to be listening to other people’s babies cry when your precious one has died” (“Debbie”, 48, p. 67). Both during the miscarriage in the hospital ward and during follow-up appointments after the miscarriage, it was difficult to be surrounded by pregnant women, and “Emily” described it as follows: “You are absolutely allergic to any other pregnant woman” (45, p. 312).

#### 3.3.2 Facilitation of partner involvement

Social support was also important to the women, and several expressed a need for their partner’s presence to share the grief and responsibility they felt they were carrying:

I would have liked my husband to be with me, at least for a night. Well, that he could stay for a night and, of course, I wanted that badly at that time. I wished that that he could stay and those would have been the perfect conditions for me to get through this… (“S”, 47, p. 12).

#### 3.3.3 Memory creation

Memory creation refers to the perception of miscarriage as losing a child and that creating memories was a way of processing the loss. This could involve pictures, memory boxes, or rituals related to the death:

I had already had this information when someone had experienced miscarriage and wanted to bury the child, and they had looked for ways how to do it. Because for me, this is life. It’s not just a mere organ that needs to be removed, thrown out. (“K”, 47, p. 11)

The memories meant a lot to the participants, and one of the women in Sanchez’s (50) study expressed the following: ‘‘I must have looked at those photos one hundred times a day for a year’’ (p. 28) about the pictures taken of the baby after the miscarriage.

Memory creation was mainly about acknowledging the child’s life, but it was also linked to personification and togetherness with the child:

We held her and touched her and cried for hours, then we had her baptized. (…) In the morning both our mothers came to the hospital with our son and the grandmas were able to hold her and nurse her and Lachlan was able to see his sister. (“Eliza”, 48, p. 68)

The quote also emphasizes the importance of allowing other family members to be involved if desired. Healthcare professionals’ handling of the child could affect this experience, and several participants expressed how important it was that healthcare professionals also treated the fetus as a child:

They treated him as if he was a living baby, telling him how perfect and beautiful he was. They treated his body with respect and explained to him what they were doing (like when they dressed him, they explained to him that they wanted him to be warm and comfortable). (“Jacquie”, 48, p. 69)

“Sam”, on the other hand, expressed how it felt when healthcare professionals neither treated nor referred to the child as a human being, which was described as very stressful: “Very much like he wasn’t a baby, to them, he was just a thing and as a parent, that’s really difficult to hear” (51, p. 870).

#### 3.3.4 Systemic limitations

The final sub-theme concerns how systemic constraints can exacerbate the experience of a miscarriage. These experiences are not necessarily related to the individual actions of healthcare professionals but connected with how the healthcare system is structured and the consequences this can have. In several studies, it was mentioned that waiting time increased the strain associated with miscarriage: “I can’t recall exactly how long I was waiting but it was certainly about an hour or two … it is a very busy area and nobody is really paying too much attention to you because they are all so busy” (“Emily”, 45, p. 311).

The systemic limitation could result from a combination of a heavy workload in the healthcare system and healthcare professionals’ approach to patients in this busy environment: “[imitating her doctor] ‘I don’t have time now. Here, do this, this, this test. See you.’ Oh no. I’m not coming back to this [doctor], ever” (“J”, 47, p. 10).

Another systemic limitation concerned the legal aspects of recognizing the child’s life. This could be, for example, that the criteria for receiving a birth certificate were not met. The lack of recognition of the child could thus intensify the grief. “Nadine” expressed the following:

We also discovered that babies under 20 weeks or 400 grams cannot have their births and deaths registered. DISGUSTING after giving birth and going through the nightmare we couldn’t even get her birth certificate. We cried and didn’t sleep or eat for days. (48, p. 70)

Several participants also pointed out how a lack of routines could also be experienced as painful. “Dorothy”, for example, experienced that healthcare professionals did not show up prepared for her consultation: “They all thought I was coming for my first scan … I think there should be something put on those folders [hospital charts], like a big sticker or something, to highlight that it wasn’t routine” (49, p. 2295).

Systemic constraints also emerged as a lack of freedom of choice or user participation. The women had a need to do what felt right for themselves and their families. Being involved in decision making was appreciated: “They let us do what we wanted to. Whether we wanted to bury, or to cremate, or just to say that we didn’t want, we didn’t want to see or to do anything with it” (“J”, 47, p. 11).

However, routines were not always in place to facilitate this: “How DARE he (the doctor) decide that I can’t hold that baby and that I won’t cope with what the baby looked like?” (“Jackie”, 44, p. 420). The quote emphasizes the importance of user involvement for the women even in a particularly difficult and vulnerable situation.

## 4 Discussion

### 4.1 Key findings

The purpose of the study was to investigate women’s experiences of their encounter with the healthcare system during and after a late miscarriage. A meta-ethnographic approach was used, synthesizing the results of eight qualitative studies. The results showed that the experiences of women with the healthcare system after a late miscarriage could be categorized into three linked main themes reflecting their need for recognition and validation of their emotional and practical needs by healthcare professionals and the health system: 1. Acknowledgment of the loss, 2. The power of language and 3. Institutional factors. The experience of acknowledgment was closely linked to how healthcare professionals communicated with the women about the loss, both verbally and nonverbally, which could be perceived as either recognizing or dismissing the loss. Finally, the women also talked about overarching aspects of the health system and health facilities that had an impact on their experience, such as being able to be shielded from other pregnant women or having the opportunity for a memorial service. Our synthesis aligns with core constructs in perinatal bereavement, trauma, and loss frameworks, such as disenfranchised grief, meaning-reconstruction, continuing bonds, and trauma-informed care.

### 4.2 Acknowledgement of the loss

For the women, it was important that their loss was recognized by healthcare professionals, and they experienced it as painful when it was not. It was important that healthcare professionals understood that not every woman thought about healthcare connected with the pregnancy loss solely as a medical procedure, but that it was more like a birth for many. It was also of great importance for the women to be met with empathy and that healthcare professionals did not judge or dismiss their feelings. This applied both to those who experienced deep grief but also to those who did not experience the loss as painful.

Studies conducted on early miscarriages have shown that having negative interactions with healthcare professionals can lead to increased discomfort related to the situation (52). The same effect was seen in stillbirths, where the negative psychological consequences for parents also can be amplified by the experience of unacknowledged grief (53). This seems also to be the case in the context of late miscarriage. Giannatiempo et al. (34) reported a similar finding in their meta-synthesis, which was linked to both healthcare professionals’ and family members’ communication and behavior. This highlights how empathetic interpersonal interactions can mitigate the negative psychological consequences of a late miscarriage. For many women, it was also painful to be left alone in their grief after discharge from the hospital and a lack of follow-up. Follow-up from the healthcare system was described by some participants as a way to get closure. Having the opportunity for both a physical and psychological follow-up after a miscarriage is appreciated by the women, and they often express a need for if it is not offered (52). Follow-up has also been shown to be effective in reducing women’s psychological distress after a miscarriage if it is carried out as a combination of psychological counseling, medical examination of the cause, and medical consultation (54).

From a bereavement perspective, this theme is directly related to the experience of disenfranchised grief – when losses are not socially or institutionally recognized (55) – amplifying distress and hindering adaptation. Our results suggest that acknowledgment of the loss by healthcare professionals reduces the risk of disenfranchised grief.

### 4.3 The power of language

Previous research has suggested that clear information, an explanation of the cause of a late miscarriage, and the opportunity to ask questions can help women feel seen and heard and counteract feelings of shame and self-blame in patients (34). This finding is supported by our study, in which several women expressed a need for regular information throughout the process, as well as for healthcare professionals to explain the medical causes for the miscarriage, to alleviate feelings of guilt. The provision of information was also closely tied to terminology in our results, with several women noting that the language was not adapted to them and that it was thus experienced as both incomprehensible and insensitive at times.

The use of terminology and empathic communication were also closely linked in our findings. Terminology is mentioned by Galeotti et al. (6) as a way to legitimize or delegitimize the experience of losing a child. For the women in our study, this largely involved calling the procedure they were about to undergo a “birth” or “stillbirth”. Several expressed that this described the experience better than the term “miscarriage” and contributed to acknowledge the loss. Furthermore, for some women, the use of terminology also had an impact on the processing of grief afterward. Lacci-Reilly et al. (4) point out that the use of words and phrases that express empathy is essential for patient-centered care. It is also highlighted in the same study that patient-centered communication can help to ease the experience of miscarriage, and that this is achieved through individualized and empathetic care, as well as clear communication. On the other hand, non-empathetic use of terminology around miscarriage can lead to an experience of unacknowledged grief (56). This illustrates how crucial the language can be as part of healthcare.

These findings reflect a meaning-reconstruction perspective in grief (57), where clear, compassionate explanations help parents make sense of their loss and reduce feelings of guilt and self-blame. Clinical terminology that recognizes birth and baby is more congruent with parents’ lived experience and may reduce traumatic appraisals. Power of language highlights how clinical communication can either validate and support meaning-making of the loss or inadvertently compound distress.

### 4.4 Institutional factors

The importance of privacy and the need to be separated from other pregnant women and babies has been highlighted in previous research (6). In our study, several women pointed out that the lack of privacy was stressful as they felt vulnerable and had a great need for shelter and care in the aftermath of the loss. Research shows that parents need privacy both because of the emotional burden, but also because of the physical symptoms and the need to discuss their medical condition with healthcare professionals without the presence of others hindering this communication (58). We found this also in our study, where the women wanted privacy as they needed to be shielded in the grieving process but also due to the fear of being a burden to other patients. A lack of facilitation of privacy can therefore be perceived as challenging for those affected, especially when they are in a phase characterized by physical and mental pain.

Furthermore, partner involvement was highlighted by several of the women in our study. They needed support from someone who was going through the same grief process and could therefore better understand and relate to their experience. Partners are considered by many women to be the most important source of support during a miscarriage (59), and to be involved in the process can thus be beneficial for the affected women and the partners themselves. The importance of partner involvement is also highlighted by Galeotti et al. (6), who point out that partners may not only react negatively to the loss, but also to the feeling of being left out and excluded from the situation.

Memory making was another aspect of institutional factors that was common in several of the studies. This included the women’s desire to spend time with the baby and objects to remember the child. Giannatiempo et al. (34) emphasize that memory making is a way to acknowledge and validate the parents’ loss of the child, but also the parents’ sense of loss of the parental role. If this is not facilitated, it can confirm the unacknowledged grief that many already experience. Furthermore, organizing a memorial service for the child can ease the grief of miscarriage as well as a way to move on (52). Facilitating memory-making can thus function as a health-promoting measure that can help parents process the loss. Opportunities for memory-making can be understood through a continuing bonds lens – maintaining an enduring connection to the child that enables adjustment without impeding functioning (60).

Finally, systemic constraints were also important factors in women’s experience during and after the miscarriage. Waiting time, legal guidelines, shared and informed decision-making were key aspects highlighted by several of the women. For many, having to wait for information and treatment while already worried about whether the baby in their womb will survive felt like a dismissal of them and their feelings. Similarly, recognition of a child through a birth and death certificate was a legal aspect that could have the same effect. When the formal requirements were not met and the parents thus did not receive these certificates, this contributed to delegitimizing the difficult experience. Several of the women in our study also expressed a need to be able to make their own decisions related to the miscarriage, with good information and support from healthcare professionals. The need for participation is emphasized in the study by Linnet Olesen et al. (61), where being included in the decision-making process was a valuable experience for the participants. Informed decision-making is also highlighted in Lacci-Reilly et al. (4), who described the need of the women to be able to make one’s own choices about the procedure and approach based on clear and thorough information from their doctor.

This domain of women’s experience maps into trauma-informed care principles, such as safety, trustworthiness, choice, collaboration, and empowerment, underscoring how environments and procedures shape bereavement trajectories after miscarriage (62).

### 4.5 Clinical implications

Our findings indicate that women have a strong need for acknowledgment, empathy, and a safe environment after a miscarriage, consistent with the principles of perinatal loss trauma informed care (62). Healthcare professionals should validate women’s experience by explicitly acknowledging the baby and the legitimacy of her emotional pain and needs. They should provide clear, timely, and understandable information, delivered sensitively, before, during, and after procedures. The woman’s preference for seeing and holding the baby and memory-making should be asked about and respected. The needs of the women and their families are complex and involve both medical and psychological care (54). Riddle et al. (14) suggest that a clinical evaluation in the context of a miscarriage should include relationship building with the patient, risk assessment, psychoeducation about grief, and the development of a plan for the way forward. According to Ryan et al. (63), examples of good routines include letting the family decide how to talk about the child and the loss and planning follow-up after discharge. In addition, good routines at an overarching level may involve giving families room for privacy, the hospital having pamphlets with important information, and the use of stickers in medical records to make it easier to pass on information about the patient to colleagues (64).

Compared to early miscarriage, late miscarriage nevertheless appears to be in a special position when it comes to recognition and healthcare needs. This is partly due to the increased attachment to the child (16, 17), while at the same time the terminology often used by healthcare providers does not prepare the parents or sufficiently validate the painful experience (4, 6, 56). As the definitions of late miscarriage and stillbirth may also overlap, it can be assumed that these two groups have similar follow-up needs. Research shows that parents who experience a stillbirth are more satisfied with the follow-up during their hospital stay (33). It may therefore be appropriate to take a closer look at this type of follow-up to be able to implement these routines in connection with late miscarriage as well.

From a Norwegian health policy perspective, it seems that less attention has been paid to the follow-up after the involuntary loss of pregnancy than to induced abortion. For example, in 2022, the national Abortion Committee was asked to make recommendations in accordance with the Abortion Act for follow-up and guidance for women undergoing termination of pregnancy (65: 29, p. 23). However, even though the committee writes in its report that the emotional reactions and the need for follow-up are regardless of the type of abortion, the committee has only focused on induced abortion and not miscarriage (65: 29, p. 23). This highlights the need for more clinically oriented knowledge about miscarriages and greater focus on this area in health policy guidelines. Knowledge about the needs of those affected by late miscarriages is particularly important to improve the healthcare they receive and should be integrated in the training of healthcare professionals. This study points to a need for changes in the healthcare system with the aim of better caring for women and families who experience a late miscarriage.

### 4.6 Methodological limitations of the included studies

The quality of included studies was assessed using CASP (43), and all studies were considered good enough to be included in the final synthesis. However, only two studies (47, 51) met criterion 6, discussing the relationship between the researcher and the participant, specifically whether the researchers critically considered their own role, bias, and influence. However, it seems that this is not as common for older studies to report, especially shorter journal articles. We therefore considered it less relevant to the overall quality, and the studies were therefore not excluded. In addition, only two studies partially met the requirements for ethical assessments (44, 48) and two others only partially met the requirements for rigid data analysis (49, 50). This meant that the studies had not gone into detail on how they had handled these issues, but that they nevertheless indicated that assessments had been made (see **Table 1**). Therefore, these studies were still considered to be of sufficient quality.

The studies span a long period of time (2001-2022), and it is reasonable to assume that much has changed in the healthcare system from when the first studies were conducted to the present day. At the same time, these are experiences that have characterized the participants during the time they experienced it, and these experiences are also an important part of the knowledge base. Furthermore, the studies have been carried out in different parts of the world, while the main emphasis is on Western countries. However, the goal of meta-ethnography is not to describe the experiences of a specific group of people, but rather the phenomenon itself in the context in which it occurs (66). We therefore do not consider it a weakness that the studies come from different contexts and are up to 24 years old but choose to mention it as it has an impact on the variation in the data material. However, most included studies were conducted in high-income Western countries and often did not disaggregate experiences of marginalized groups (e.g., indigenous people, ethnic minorities, migrants/refugees, gender minorities). This limits the generalizability of our synthesis. Future research should prioritize studies in non-Western and low- and middle-income countries and within marginalized populations, to guide culturally informed, equity-oriented bereavement care after miscarriage.

Moreover, a limitation of several of the included studies may be the representativeness of the sample. Purposive sampling is often used in qualitative studies to ensure depth and variation in the data material, but does not necessarily guarantee generalizable results (67). In addition, the characteristics of those who agree to participate can have an impact on the results, for example, those who have positive experiences are more likely to participate in qualitative studies (68). This may have introduced a bias into the included data material.

### 4.7 Strengths and limitations of this study

A strength of the meta-ethnographic approach is its ability to handle heterogeneity in data sets, and to provide a more interpretive description of the phenomenon in question (66, 69). We therefore believe that this method is well-suited to addressing our research questions. However, it is often pointed out that this approach provides little guidance regarding the selection of studies and quality assessment (69). We have therefore discussed the selection process with a university librarian, in addition to examining the process in other studies to increase our understanding. For the same reason, we have also chosen to use an external quality assessment (43). We selected meta-ethnography for its interpretive strength in generating higher-order constructs across qualitative studies. Alternative mixed synthesis approaches, e.g., narrative synthesis (70) or critical interpretive synthesis (71) could also have been appropriate and might have yielded additional theoretical insights. Future reviews could apply these approaches to test the robustness of our model.

Our meta-ethnography included eight individual studies. Noblit and Hare’s (35) original description estimated the appropriate number of studies to be between two and six, but later articles have suggested up to 40 included studies (72). At the same time, theoretical saturation is considered to be more important than the number of included studies within a meta-ethnographic framework (73). This is achieved when new data do not add anything to the theory developed in the study. However, Toye et al. (74) point out that whether or not theoretical saturation has been achieved is a subjective assessment, and that there is always a degree of uncertainty as to whether the balance between robust and diluted data has been achieved. We concluded that the meta-ethnography has reached a certain theoretical saturation, although it would have been advantageous to have more data. On the other hand, there were few published studies that focused solely on late miscarriage, which led to a limited number of studies that was possible to include. There were also several studies we did not include because they did not provide exact weeks for the participants’ pregnancy loss. In addition, there was variation in the terms used in the research articles to describe miscarriage. This may have led us to exclude relevant studies because they used terms commonly associated with other types of pregnancy loss.

### 4.8 Further research

Although there is a number of studies in this area of miscarriage, there are still knowledge gaps in this field. For example, a scoping review from 2017 looked at the effect of interventions to reduce stress in pregnant women with a history of miscarriage (75). Out of more than 4,000 articles that were screened, none met the inclusion criteria, which points to a need to gather and systematize existing knowledge on a topic that has a clear impact on the lives of those affected. In addition, research is needed on how siblings, grandparents and other family members are affected by a miscarriage, with the aim of developing more comprehensive healthcare services. Research in Norway will also be important to better understand this phenomenon and determine whether similar experiences occur in the Norwegian healthcare system.

## 5 Conclusion

This meta-synthesis on women’s experiences with the healthcare system after a late miscarriage helps to shed light on 1. the need for acknowledgment of the loss, 2. the power of language, and 3. the impact of institutional factors. Our findings align with existing research on experiences following early miscarriage and stillbirth. The findings also point to changes that may be necessary to improve the healthcare system after late miscarriage. However, further research is needed to better understand the impact of late miscarriage on women and their families and to develop improved guidelines for follow-up care.

## Data Availability

The original contributions presented in the study are included in the article/**Supplementary Material**. Further inquiries can be directed to the corresponding author.
