# Supplementary material for: Women’s experiences with the healthcare system during and after a late miscarriage: a qualitative meta-synthesis
Source: Front Psychiatry. 2026 Jan 5;16:1673215. doi: 10.3389/fpsyt.2025.1673215 (PMC12812685; doi:10.3389/fpsyt.2025.1673215)
Supplement: Supplementary file 1 [file Table1.docx]

**Supplementary material**

**Table S1** *Search string from PsycInfo*

| Theme | Methodology |
| --- | --- |
| pregnancy loss*.ti,ab,id. OR miscarr*.ti,ab,id. OR perinatal loss*.ti,ab,id. OR baby loss*.ti,ab,id. OR perinatal death*.ti,ab,id. OR fetal loss*.ti,ab,id. OR fetal death*.ti,ab,id. OR intrauterine death*.ti,ab,id. OR spontaneous abortion*.ti,ab,id. | (interview* OR experience*).af. OR qualitative.tw. |

**Table S2** *Quality assessment of the included studies using CASP*

| Assessment criteria | Corbet-Owen & Kruger (2001) | Cullen et al (2018) | Ekelin et al (2008) | Kukulskiené & Žemaitiené, (2022) | Lee (2012) | Mulvihill & Walsh (2014) | Sanchez (2001) | Smith et al (2020) |
| --- | --- | --- | --- | --- | --- | --- | --- | --- |
| 1. Was there a clear statement of the aims of the research? | Yes | Yes | Yes | Yes | Yes | Yes | Yes | Yes |
| 2. Is a qualitative methodology appropriate? | Yes | Yes | Yes | Yes | Yes | Yes | Yes | Yes |
| 3. Was the research design appropriate to address the aims of the research? | Yes | Yes | Yes | Yes | Yes | Yes | Yes | Yes |
| 4. Was the recruitment strategy appropriate to the aims of the research? | Yes | Yes | Yes | Yes | Yes | Yes | Yes | Yes |
| 5. Were the data collected in a way that addressed the research issue? | Yes | Yes | Yes | Yes | Yes | Yes | Yes | Yes |
| 6. Has the relationship between researcher and participants been adequately considered? | Partly | Partly | Partly | Yes | Partly | no | Partly | Yes |
| 7. Have ethical issues been taken into consideration? | Partly | Yes | Yes | Yes | Partly | Yes | Yes | Yes |
| 8. Was the data analysis sufficiently rigorous? | Yes | Yes | Yes | Yes | Yes | Partly | Partly | Yes |
| 9. Is there a clear statement of findings? | Yes | Yes | Yes | Yes | Yes | Yes | Yes | Yes |
| 10. How valuable is the research? ^(a^ | Yes | Yes | Yes | Yes | Yes | Yes | Yes | Partly |

^a^ We have answered this question respectively with "yes", "no" or "partially" based on whether we have considered the research valuable, not the degree how valuable it is (as the question suggests).
